# EWS Knockdown and Taxifolin Treatment Induced Differentiation and Removed DNA Methylation from p53 Promoter to Promote Expression of Puma and Noxa for Apoptosis in Ewing’s Sarcoma

**DOI:** 10.4236/jct.2014.512114

**Published:** 2014-10-28

**Authors:** Mohammad Motarab Hossain, Swapan Kumar Ray

**Affiliations:** Department of Pathology, Microbiology, and Immunology, University of South Carolina School of Medicine, Columbia, SC, USA

**Keywords:** Apoptosis, Differentiation, Ewing’s Sarcoma, EWS shRNA, p53 Promoter, Taxifolin

## Abstract

Ewing’s sarcoma is a pediatric tumor that mainly occurs in soft tissues and bones. Malignant characteristics of Ewing’s sarcoma are correlated with expression of EWS oncogene. We achieved knockdown of EWS expression using a plasmid vector encoding EWS short hairpin RNA (shRNA) to increase anti-tumor mechanisms of taxifolin (TFL), a new flavonoid, in human Ewing’s sarcoma cells in culture and animal models. Immunofluorescence microscopy and flow cytometric analysis showed high expression of EWS in human Ewing’s sarcoma SK-N-MC and RD-ES cell lines. EWS shRNA plus TFL inhibited 80% cell viability and caused the highest decreases in EWS expression at mRNA and protein levels in both cell lines. Knockdown of EWS expression induced morphological features of differentiation. EWS shRNA plus TFL caused more alterations in molecular markers of differentiation than either agent alone. EWS shRNA plus TFL caused the highest decreases in cell migration with inhibition of survival, angiogenic and invasive factors. Knockdown of EWS expression was associated with removal of DNA methylation from p53 promoter, promoting expression of p53, Puma, and Noxa. EWS shRNA plus TFL induced the highest amounts of apoptosis with activation of extrinsic and intrinsic pathways in both cell lines in culture. EWS shRNA plus TFL also inhibited growth of Ewing’s sarcoma tumors in animal models due to inhibition of differentiation inhibitors and angiogenic and invasive factors and also induction of activation of caspase-3 for apoptosis. Collectively, knockdown of EWS expression increased various anti-tumor mechanisms of TFL in human Ewing’s sarcoma in cell culture and animal models.

## 1. Introduction

Tumors of the Ewing’s sarcoma family (Ewing’s sarcoma, Askin’s tumor, and primitive neuroectodermal tumor) commonly occur in soft tissues and bones in children and young adults [1]. Ewing’s sarcoma is the second most common malignant bone tumor that accounts for 10% - 15% of all primary bone tumors in pediatric and adolescence patients [1]. Ewing’s sarcoma is often a metastatic malignancy at the time of diagnosis. Current therapeutic strategies include local surgery and radiotherapy in conjunction with systemic chemotherapy followed by stem cell transplantation. However, therapeutic inefficacy in most cases accounts for approximately less than 20% long-term survival of metastatic Ewing’s sarcoma patients [2]. So, there is an urgent need for development of new and effective therapeutic strategies for treating these childhood tumors.

The Ewing’s sarcoma (EWS) gene on human chromosome 22q12 is involved in formation of a wide variety of solid tumors including Ewing’s sarcoma [3]. This gene encodes the multifunctional EWS protein that includes an N-terminal transcriptional activation domain and a C-terminal RNA-binding domain. Chromosomal translocations between this gene and various genes encoding transcription factors result in the production of chimeric proteins that are involved in tumorigenesis. These chimeric proteins usually consist of the N-terminal transcriptional activation domain of this protein fused to the C-terminal DNA-binding domain of the transcription factor protein. In Ewing’s sarcoma, the chomosomal translocation t(11; 22)(q24; q12) generates a fusion of the 5’ transcriptional activation domain of EWS gene with the 3’ DNA-binding domain of Friend leukemia integration 1 (FLI1) gene resulting in the fusion oncoprotein EWS-FLI1, which acts as an aberrant transcriptional activator with strong transforming capabilities [4]. About 80% - 90% of Ewing’s sarcomas contain the chromosomal translocation t(11; 22) (q24; q12). Stable expression of anti-sense EWS-FLI1 cDNA and dominant-negative inhibition of EWS-FLI1 cDNA resulted in growth reduction and provided evidence of EWS/FLI-l involvement in proliferation of Ewing’s sarcoma cells [5]. However, therapeutic strategies to eliminate or inactivate EWS-FLI1 have not yet been successfully translated to clinics [6]. Notably, EWS-FLI1 has poor solubility that makes it very difficult to directly analyze this molecule *in vitro*. However, anti-sense oligonucleotide directed against the EWS part of EWS-FLI1 is known to cause tumor inhibitory effect *in vivo* [7]. In Ewing’s sarcoma, EWS co-exists with EWS/FLI that is known to bind EWS and interfere with EWS driven transcription, splicing, and maintenance of genomic stability [8] [9]. EWS regulates the expression of cyclin D1, which controls cell cycle transition at G1/S phase. In addition, EWS regulates the DNA damage-induced alternative splicing of genes involved in DNA repair and stress response and EWS is needed for cell survival following DNA damage [10]. In fact, EWS knockout mice are hypersensitive to ionizing radiation and prone to premature cellular senescence [11]. A very recent study reported that knockdown of EWS expression due to transfection of EWS short hairpin RNA (shRNA) plasmid increased neuronal phenotype and inhibited oncogenic transformation in Ewing’s sarcoma cell lines [12]. Therefore, EWS is now considered to be the best target for anti-tumor therapy in Ewing’s sarcoma. Introduction of a shRNA into the cells through transfection of a plasmid vector encoding a gene-specific shRNA is a very powerful technique to knockdown a particular mRNA molecule and subsequently the protein level of the targeted gene, as we have also demonstrated recently [13]. EWS shRNA plasmid transfection can thereby be an effective strategy for knockdown of EWS expression to induce differentiation, inhibit migration and proliferation, and promote apoptosis in human Ewing’s sarcoma cells in culture as well as in animal models.

Flavonoids are polyphenolic compounds that are ubiquitous in plants. Plant polyphenolic compounds exhibit several positive effects on human health because of their proven anti-microbial, anti-viral, and anti-tumor properties. The anti-tumor properties of dietary flavonoids are widely recognized [14]. Taxifolin (TFL), which is a pentahydoxyflavanone, is predominantly found in milk thistle [15] and slightly in red onion [16]. A recent study indicates that TFL is not mutagenic [17]. The chemopreventive effects of TFL have been documented in colon cancer cells [18]. A previous study has shown that TFL in a dose-dependent manner inhibits the growth of ovarian cancer cells [19]. A more recent study suggests that TFL targets epidermal growth factor receptor (EGFR) and phosphoinositide 3-kinase (PI3K) for chemoprevention of the UV-induced skin carcinogenesis [20]. Two different studies demonstrate that TFL induces apoptosis in prostate cancer cells [21] [22]. But the molecular mechanisms and targets of TFL for its anti-tumor activities in Ewing’s sarcoma have not yet been investigated.

In this investigation, we performed knockdown of EWS expression using EWS shRNA plasmid for potentiation of anti-tumor properties of TFL in two human Ewing’s sarcoma cell lines, SK-N-MC and RD-ES, in culture and animal models. We decided to use these two cell lines to determine the efficacy of our new combination therapy in diverse Ewing’s sarcoma cells in culture and animal models. We examined whether our new combination therapy could induce cell differentiation and inhibit cell migration, angiogenesis, and invasion leading to apoptosis.

## 2. Materials and Methods

### 2.1. Cell Lines and Culture Conditions

Human Ewing’s sarcoma SK-N-MC and RD-ES cell lines were purchased from the American Type Culture Collection (ATCC, Manassas, VA). Cells were maintained in 75-cm^2^ flasks containing 1× RPMI 1640 supplemented with 10% fetal bovine serum (FBS) and 1% penicillin and 1% streptomycin (GIBCO/BRL, Grand Island, NY) in a fully-humidified incubator containing 5% CO_2_ at 37°C. Both control shRNA plasmid (encoding scrambled shRNA sequence) and EWS shRNA plasmid were purchased from Santa Cruz Biotechnology (Santa Cruz, CA). TFL and mithramycin were obtained from Sigma Chemical (St. Louis, MO) and dissolved in dimethyl sulfoxide (DMSO) to make stock solutions and aliquots of stock solutions were stored at −20°C until ready to use.

### 2.2. Immunofluorescence Microscopy to Examine Expression of EWS

Laser scanning confocal immunofluorescence microscopy was performed according to our previously described method [23] with slight modification. Briefly, cells were grown on the poly-D-lysine chamber slides (BD Biosciences, Bedford, MA) at 37°C in presence of full-humidity and 5% CO_2_ for 24 h. Cells on the slides were washed twice in phosphate-buffered saline (PBS), pH 7.4, (without Ca^2+^ and Mg^2+^) followed by fixation in 4% formaldehyde in PBS for 30 min at room temperature. After fixation, cells were washed in PBS and made permeable with 0.25% Triton X-100 in PBS for 10 min. Cells were washed and non-specific binding were blocked by 3% bovine serum albumin (BSA) in PBS for 10 min. Primary IgG antibody against EWS (Santa Cruz Biotechnology, Santa Cruz, CA) was diluted (1:200) in 1% BSA in PBS. After the incubation with primary IgG antibody for 1 h at room temperature, cells were washed twice with PBS and stained with fluorescein isothocyanate (FITC) conjugated anti-rabbit secondary IgG antibody (Jackson Immunoresearch, West Grove, PA) in 1% BSA in PBS for 1 h at room temperature and washed again with PBS. To counter stain the nucleus, cells were then incubated with 300 nM 4’,6-diamidino-2-phenylindole (DAPI) (Invitogen, Eugene, OR) for 5 min and washed four times before mounting with Dabco 33-LV (Sigma Chemical, St. Louis, MO). The nuclear stain DAPI could strongly bind to A-T rich regions in DNA to show blue fluorescence. Fluorescence images were captured using Zeiss LSM 510 META confocal microscope (Zeiss, Germany) and analyzed by Zeiss LSM image browser software.

### 2.3. Flow Cytometry to Monitor Expression of EWS

Adherent Ewing’s sarcoma cells were detached from culture flasks by incubating with 0.02% EDTA (Sigma Chemical, St. Louis, MO) in PBS and then dissociated into monodispersed cells. Cells (1 × 10^5^) were collected and suspended in 100 μl of 1× flow cytometry buffer (1% BSA and 0.1% NaN_3_ in PBS, filtered through the 0.22 μm filter unit) containing an FcBlocker (eBioscience, San Diego, CA) and incubated at 4°C for 15 min. Primary IgG antibody against EWS (Santa Cruz Biotechnology, Santa Cruz, CA) was diluted (1:200) in 1% BSA in PBS and then added to the cell suspension. Parallel staining was also performed with rabbit IgG (1:200) (Jackson Immunoresearch, West Grove, PA) as an isotype matched control for EWS. After incubation at 4°C for 30 min, cells were washed twice with the 1× flow cytometry buffer. Cells were then incubated in 100 μl of 1× flow cytometry buffer containing FITC-conjugated goat anti-rabbit secondary IgG antibody (1:200) at 4°C for 30 min. Subsequently, cells were washed as above, fixed in 2% paraformaldehyde in PBS and then transferred into a 5-ml polystyrene round-bottom tube capped with a cell-strainer 183 cap (BD Biosciences, Franklin Lakes, NJ). Antibody-bound cells were then analyzed on Epics XL-MCL Flow Cytometer (Beckman Coulter, Fullerton, CA) using CellQuest software (BD Biosciences, Bedford, MA) and also FlowJo software (Tree Star, Ashland, OR).

### 2.4. Transfection with EWS shRNA Plasmid and Treatment with TFL or Mithramycin

Human Ewing’s sarcoma SK-N-MC and RD-ES cell lines were grown at 37°C with 5% CO_2_ and full-humidity. Both cell lines were transfected with scrambled shRNA plasmid or EWS shRNA plasmid, treated with TFL alone, combination of EWS shRNA plasmid and TFL or mithramycin in medium containing 2% FBS. It should be noted that the cells were transfected with the plasmid (0.5 μg/ml) in 6-well dishes using Lipofectamine 2000 transfection reagent (Invitrogen, Carlsbad, CA) according to the manufacturer’s instructions and incubated at 37°C with 5% CO_2_ and full-humidity. After 6 h, transfection reagent was replaced by fresh medium containing 2% FBS. The old medium was replaced by fresh medium at 18 h and again at 48 h without and with TFL or mithramycin and incubated for another 24 h.

### 2.5. The 3-(4,5-Dimethylthiazol-2-yl)-2,5-Diphenyltetrazolium Bromide (MTT) Assay

The effects of scrambled shRNA plasmid, EWS shRNA plasmid, TFL, and combination of EWS shRNA plasmid and TFL or mithramycin on the viability of human Ewing’s sarcoma SK-N-MC and RD-ES cell lines were determined by the MTT assay [24]. Both cell lines were subjected to scrambled shRNA plasmid (0.5 μg/ml) or EWS shRNA plasmid (0.5 μg/ml) transfection, TFL (25, 50, and 100 μM) treatment, combination of EWS shRNA plasmid (0.5 μg/ml) transfection and TFL (25, 50, or 100 μM) treatment, and combination of EWS shRNA plasmid (0.5 μg/ml) transfection and mithramycin (0.25, 0.5, or 1 μM) treatment. After transfection, treatment, or both, medium was discarded and cells were washed with PBS and subsequently incubated with MTT (0.2 mg/ml) in 100 μl of fresh medium for 2 h. Then, 2-propanol (200 μl) was added to dissolve MTT formazan crystals and absorbance was measured at 570 nm in a plate reader (BioTek, Winooski, VT). Residual cell viability was calculated as the percentage of viable cells in total population.

### 2.6. Reverse Transcription-Polymerase Chain Reaction (RT-PCR) to Examine Expression of EWS mRNA

The semi-quantative RT-PCR experiment, as we described recently [25], was used to monitor the levels of expression of EWS mRNA in human Ewing’s sarcoma SK-N-MC and RD-ES cell lines. Both cell lines were subjected to no treatment, scrambled shRNA plasmid (0.5 μg/ml) transfection, EWS shRNA plasmid (0.5 μg/ml) transfection, 100 μM TFL treatment, and combination of EWS shRNA plasmid transfection and TFL treatment in medium containing 2% FBS. Total RNA was isolated from the cells using TRIzol reagent (Invitrogen, Carlsbad, CA). We used the following primer sequences for PCR amplifications of EWS gene (forward: 5’-ATG GCG TCC ACG GAT TAC AGT ACC −3’ and reverse: 5’-CGG CTG TGT AGA GGA ATA GCT GGT AGG-3’) and GAPDH gene (forward: 5’-ATG GGG AAG GTG AAG GTC GG-3’ and reverse: 5’-AGA CGC CAG TGG ACT CCA CGA CG-3’). Total RNA (300 ng) of each sample was used to synthesize cDNA using SuperScript one-step RT-PCR kit (Invitrogen, Carlsbad, CA) on a thermal cycler (Eppendorf, Westbury, NY) at 50°C for 30 min and inactivation of reverse transcriptase at 94°C for 3 min followed by 30 cycles of amplification (denaturation of template at 94°C for 15 sec, annealing of primers at 55°C for 30 sec, and extension of primers at 72°C for 1 min) and final extension at 72°C for 10 min. The RT-PCR products were resolved by electrophoresis on 1% agarose gels, stained with ethidium bromide (1 μg/ml), destained the background in water, and visualized and photographed the gels using UVDI Compact Digimage System (Major Science, Saratoma, CA). Expression of GAPDH, a housekeeping gene, was used as an internal control.

### 2.7. Protein Extraction

For protein extraction, Ewing’s sarcoma cells or tissues were homogenized in ice-cold homogenization solution (50 mM Tris-HCl, pH 7.4, 320 mM sucrose, 0.1 mM phenylmethylsulfonyl fluride, and 1 mM EDTA), transferred to eppendorf tubes, and subjected to sonication gently in micro-ultrasonic cell disruptor (Kontes, Vinel- and, NJ). The lysates were centrifuged at 12,000 rpm for 10 min at 4°C and the supernatants were collected. The protein concentrations in the supernatants were measured using Coomassie plus protein assay reagents (Pierce Biotechnology, Rockford, IL). All the samples were divided into small aliquots and stored at −20°C until they were used.

### 2.8. Western Blotting Using Specific Antibodies

Each protein sample (10 μg) was mixed with Laemmli buffer and denatured in boiling water for 5 min. Then, all protein samples were loaded onto the precast 4% - 20% polyacrylamide gradient gels (Bio-Rad Laboratories, Hercules, CA) for sodium dodecyl sulfate-polyacrylamide gel electrophoresis (SDS-PAGE). After the SDS-PAGE, the resolved protein samples from the gels were electroblotted to the polyvinylidene fluoride (PVDF) membranes (Millipore, Bedford, MA). The non-specific binding sites on the PVDF membranes were blocked with 5% non-fat dry milk for 1 h at room temperature. The primary IgG antibody against *β*-actin (clone AC-15) was purchased from Sigma Chemical (St. Louis, MO) and primary IgG antibodies against EWS, E-cadherin, Notch-1, inhibitor of differentiation 2 (ID2), human telomerase reverse transcriptase (hTERT), proliferating cell nuclear antigen (PCNA), p-Akt (Thr 308), p65 nuclear factor-kappa B (NF-*κ*B), vascular endothelial growth factor (VEGF), basic fibroblast growth factor (b-FGF), matrix metalloproteinase-2 (MMP-2), MMP-9, p53, Puma, Noxa, caspase-8, tBid, Bax, Bcl-2, Cox4, cytochrome c, Smac/Diablo, apoptosis-inducing factor (AIF), calpain, caspase-3, spectrin breakdown product (SBDP), and inhibitor of caspase-activated DNase (ICAD) were obtained from Santa Cruz Biotechnology (Santa Cruz, CA). The PVDF membranes or blots were incubated overnight at 4°C for shaking on a rocker with an appropriate dilution of a primary IgG antibody followed by three times washing in a washing buffer (20 mM Tris-HCl, pH 7.6, 137 mM NaCl, 0.1% Tween-20). Then, the blots were incubated with an appropriate alkaline horseradish peroxidase (HRP)-conjugated secondary IgG antibody for 1 h followed by three times washing in the washing buffer. Alkaline HRP-conjugated anti-rabbit and anti-mouse secondary IgG antibodies were purchased from Biomeda (Foster City, CA) and anti-goat secondary IgG was from Santa Cruz Biotechnology (Santa Cruz, CA). Specific protein bands were detected by incubation of the blots for 5 min at room temperature with Immun-Star^™^ HRP Lumino/Enhancer (Bio-Rad Laboratories, Hercules, CA) followed by autoradiography using BIOMAX XAR films (Kodak, Rochester, NY). Autoradiograms were scanned on EPSON scanner using Photoshop software (Adobe Systems, Seattle, WA). All experiments were performed in triplicates.

### 2.9. *In Situ* Methylene Blue Staining for Morphological Features of Differentiation

After transfection of human Ewing’s sarcoma SK-N-MC and RD-ES cells with scrambled shRNA plasmid or EWS shRNA plasmid, cells were cultured in 6-well dishes for 72 h. The transfected cells were washed twice with ice-cold PBS in the culture plate followed by fixation of cells with 2 ml of ice-cold 95% (v/v) ethanol for 5 min. After fixation, we aspirated ethanol, washed the cells twice with PBS, and then stained with 2 ml of ice-cold 0.2% (v/v) methylene blue solution (prepared in 50% ethanol) for 20 sec. After staining, the cells were washed with distilled water and air dried before taking photograph under the light microscope. The dimensions of the cells (length and width) and length of extensions were measured (n = 100) using ImagePro Plus software version 4.5.1.29 (Media Cybernetics, Silver Spring, MD).

### 2.10. *In Vitro* Cell Migration Assay

*In vitro* cell migration assay, as we reported recently [26], was performed to determine the effects of EWS shRNA plasmid or TFL alone and combination of both on the migratory property of human Ewing’s sarcoma SK-N-MC and RD-ES cells. The migration assay was carried out in 6-well transwell inserts of polycarbonate membrane with 8.0 μm pore size (Corning, Lowell, MA). The transwell inserts were coated with matrigel (BD Biosciences, San Jose, CA) of final concentration of 1.0 mg/ml in an ice-cold serum-free medium and allowed to dry at 37°C for 4 h. After the treatments, cells were trypsinized and washed twice with serum-free medium. Then, cell suspension (1 × 10^5^ cells in 500 μl) from each sample was added to each transwell insert in triplicate. Cells were incubated at 37°C in presence of 5% CO_2_ for 48 h and the membranes were collected and stained with DIFF quick stain kit (IMEB, San Marcos, CA). The cells that migrated to undersurface of the membrane were observed using light microscope, photographed, and counted in 10 randomly selected microscopic fields.

### 2.11. Sodium Bisulfite Treatment of Genomic DNA and Methylation-Specific PCR (MSP) Analysis of p53 Promoter Region

We used standard MSP [27] [28] to determine the comparative efficacy of our treatments and the known demethylating agent 5’-Aza-2-deoxyCytosine (AzaC) [29] in changing status of methylation of DNA at the tumor suppressor p53 promoter region in Ewing’s sarcoma SK-N-MC and RD-ES cells. After the treatments, genomic DNA from the cells was purified using TRIzol reagent (Invitrogen, Carlsbad, CA) according to manufacturer’s instructions. Genomic DNA was treated with sodium bisulfite to convert unmethylated cytosine to thymidine using CpGenome Universal DNA Modification kit (Chemicon, Temecula, CA) according to the manufacturer’s instructions. The bisulfite-treated DNA was used as template for MSP amplification of the human p53 promoter region using MSP primers specific for either methylated (M pair) or unmethylated (U pair) DNA [27]. MSP primers (left M primer: 5’-ATT TTA TTT TTT TTG TTT TTT TCG G-3’; right M primer: 5’-AAC AAC TAC CTA CTC CCT AAA CGA T-3’; left U primer: 5’-TTT TAA TTT TAT TTT TTT TGT TTT TTT TG-3’; and right U primer: 5’-AAC AAC TAC CTA CTC CCT AAA CAA T-3’) were designed using the MethPrimer program (http://www.urogene.org/methprimer/index.html). Amplification with M, U, and both primers indicated methylation, unmethylation, and partial methylation, respectively.

### 2.12. *In Situ* Wright Staining for Morphological Features of Apoptosis

After the treatments, both adherent and non-adherent SK-N-MC and RD-ES cells were spun down at 3500 rpm for 10 min. Cells were washed with PBS, fixed, and stained with HEMA3 stain according to the manufacturer’s instruction (Fisher Scientific, Kalamazoo, MI). Cells were allowed to dry after the staining and the morphological features of the cells were observed under the light microscope and digital pictures were captured. All the experiments were conducted in triplicates and only representative pictures were presented.

### 2.13. Annexin V Staining Followed by Flow Cytometry for a Biochemical Feature of Apoptosis

After the treatments, both adherent and non-adherent SK-N-MC and RD-ES cells were collected in 15-ml tubes and washed twice with 10 ml of PBS. Cells were stained with Annexin V-fluorescein isothiocyanate (FITC)/propidium iodide (PI), processed according to manufacturer’s instructions (BD Bioscineces, SD, CA), and then analyzed on Epics XL-MCL Flow Cytometer (Beckman Coulter, Fullerton, CA), as we reported recently [13]. Annexin V-FITC negative and PI positive cells were considered as mechanically injured (quadrant A1), both Annexin V-FITC and PI positive cells were considered as late necrotic (quadrant A2), both Annexin V-FITC and PI negative cells were considered as normal (quadrant A3), and Annexin V-FITC positive and PI negative cells were considered as early apoptotic (quadrant A4). Flow cytometry detected the Annexin V-FITC positive cells with externalization of membrane phospholipids, an early biochemical feature of apoptosis. All the experiments were conducted in triplicates and only representative pictures were shown. The Annexin V-FITC stained apoptotic cells were analyzed for statistical significance.

### 2.14. Development of Ewing’s Sarcoma Tumors and Treatments

We conducted all animal studies in strict accordance with the recommendations in the “Guide for the Care and Use of Laboratory Animals” of the National Institutes of Health and also with the approval from the Institutional Animal Care and Use Committee (IACUC) of the University of South Carolina (Columbia, SC). We performed all surgical procedures under anesthesia condition and made all efforts to minimize animal suffering. For using the cells in development of Ewing’s sarcoma xenografts, about 80% confluent cultures of SK-N-MC and RD-ES cells were separately harvested, counted, and suspended in an equal volume of high-concentrated matrigel (BD Biosciences, San Jose, CA). We used 100 μl of cell suspension (5 × 10^6^ cells) in matrigel to inject subcutaneously (under the dorsal skin) in 6-week old nude mice (Charles River Laboratories, Wilmington, MA). The animals were left for 1 week without any treatment for uniform development of tumors with approximate volume of 150 to 250 mm^3^. The animals were then divided into 4 groups of 6 mice in each group. Tumor bearing mice were injected carefully at the tumor site with scrambled shRNA plasmid (50 μg DNA/injection/mouse), EWS shRNA plasmid (50 μg DNA/injection/mouse), TFL (20 μg/injection/mouse), or EWS shRNA plasmid (50 μg DNA/injection/mouse) plus TFL (20 μg/injection/mouse) on alternate days for 2 weeks. At the end of treatments, all animals were anesthetized with intraperitoneal injection of a mixture of ketamine (50 mg/kg) and xylazine (35 mg/kg) and photographed. Solid tumors were surgically removed. Tumor volume was determined by direct measurement with calipers using a previously reported formula [30]. Tumors from all treatments were photographed. Tumors were used immediately for some experiments or stored at −80°C for other experiments.

### 2.15. Histopathological Changes in Tumor Sections

After completion of treatment schedule, mice were sacrificed and Ewing’s sarcoma tumors were surgically collected and washed with PBS (pH 7.4). Tumors were immediately frozen (−80°C) in Optima Cutting Temperature media (Fisher Scientific, Suwanee, GA) and 10 μm sections were cut with Microm HM 505N (Labequip, Markham, Ontario). Tumor sections were subjected to conventional hematoxylin and eosin (H & E) staining. The H & E stained tumor sections were examined under Olympus BX53 Versatile System microscope (Olympus America, Center Valley, PA) for histopathological changes such as decrease in cell proliferation and increase in cell death. The H & E stained tumor sections were then photographed.

### 2.16. Statistical Analysis

The results from some of the experiments were analyzed for statistical significance using Minitab 16 statistical software (Minitab, State College, PA). Data were expressed as mean ± standard deviation (SD) of separate experiments (n ≥ 3) and compared by one-way analysis of variance (ANOVA) followed by the Fisher’s post-hoc test. Difference between control (CTL) or scrambled shRNA group and a treatment group was considered significant at *P* < 0.05.

## 3. Results

### 3.1. *In Situ* Immunofluorescence Microscopy and Flow Cytometry to Examine Expression of EWS

**Figure 1:**
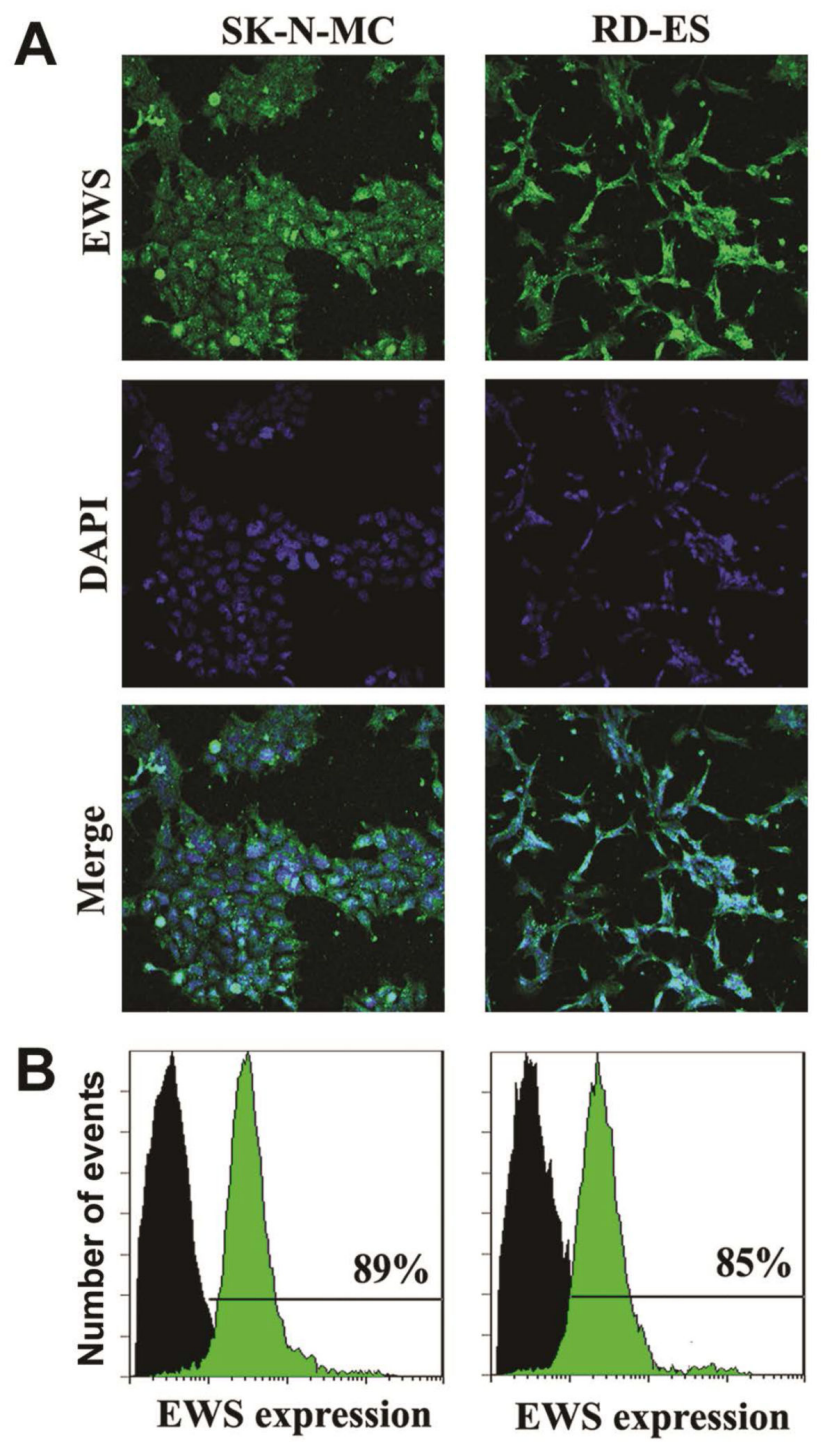
Detection and determination of expression of EWS in human Ewing’s sarcoma SK-N-MC and RD-ES cell lines. (A) Representative *in situ* immunofluorescence staining to show the expression of EWS (FITC, green) and nuclei (DAPI, blue) in the cells. (B) Immunofluorescence staining of the cells followed by flow cytometry to determine the levels of expression of EWS (FITC, green) when compared with the control cells (without staining, black).

Presence of EWS gene is responsible of expression of EWS protein in human Ewing’s sarcoma cell lines. Two different methods were used to examine the expression of EWS in two different human Ewing’s sarcoma SK-N-MC and RD-ES cell lines (Figure 1). Cells were immunostained with FITC (green fluorescence) conjugated EWS antibody. The *in situ* expression of EWS was detected by laser scanning confocal immunofluorescence microscopy, which demonstrated expression of EWS protein at high levels in both SK-N-MC and RD-ES cell lines (Figure 1A). Cell nuclei were stained with DAPI (blue fluorescence). Thereafter, we used flow cytometry to quantify the levels of expression of EWS protein in these cell lines (Figure 1B). Our results from flow cytometric analysis indicated that levels of expression of EWS were 89% and 85% in SK-N-MC and RD-ES cell lines, respectively (Figure 1B). Thus, our results (Figure 1) demonstrated and determined the relative expression of EWS protein in SK-N-MC and RD-ES cell lines, both of which were used in all subsequent experiments.

### 3.2. Combination of EWS shRNA Plasmid Transfection and TFL Treatment Decreased Cell Viability

**Figure 2:**
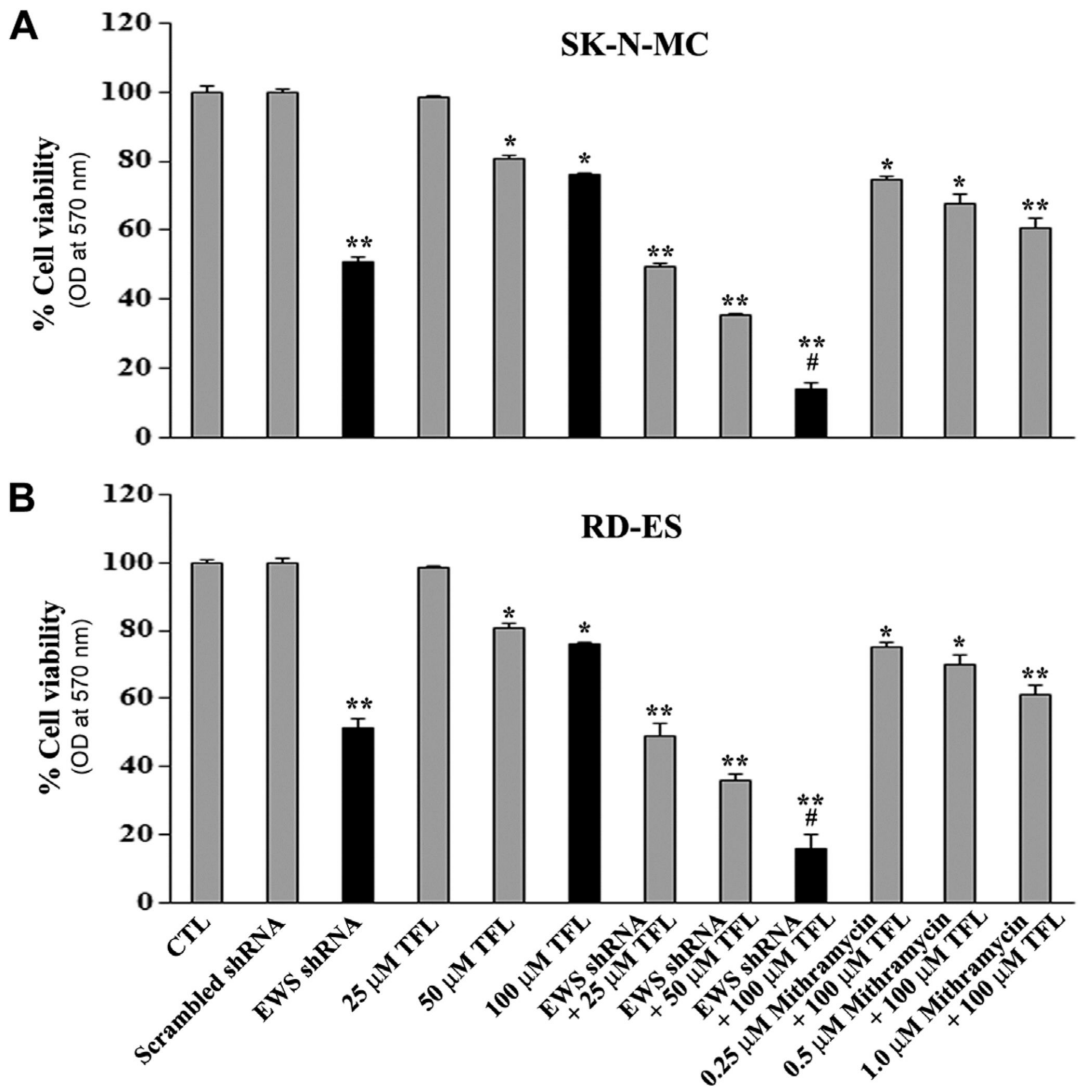
Determination of residual cell viability. Amounts of residual cell viability of Ewing’s sarcoma (A) SK-N-MC cells and (B) RD-ES cells subjected to no treatment control (CTL), scrambled shRNA plasmid (0.5 μg/ml) transfection for 72 h, EWS shRNA plasmid (0.5 μg/ml) transfection for 72 h, TFL (25, 50, or 100 μM) treatment for 24 h, EWS shRNA palsmid transfection for 48 h + TFL treatment for last 24 h, and EWS shRNA plasmid transfection for 48 h + mithramycin (0.25, 0.5, or 1.0 μM) treatment for last 24 h. Mean values (n = 3) were shown and significant difference between two values was indicated by *P < 0.05 or **P < 0.01 (where monotherapy was compared with CTL) and ^#^*P* < 0.01 (where combination therapy was compared with monotherapy).

Thereafter, we examined whether EWS shRNA plasmid transfection and TFL treatment might work together to reduce viability of human Ewing’s sarcoma SK-N-MC and RD-ES cell lines (Figure 2). We subjected SK-N-MC and RD-ES cell lines to scrambled shRNA plasmid (0.5 μg/ml) transfection, EWS shRNA plasmid (0.5 μg/ml) transfection, treatment with different concentrations of TFL (25, 50, and 100 μM), and combination of EWS shRNA plasmid transfection and TFL treatment (Figure 2). The MTT assay showed that the scrambled shRNA plasmid transfection did not have any effect on cell viability whereas EWS shRNA plasmid transfection and TFL treatment alone and in combination inhibited cell viability at varying degrees. We found that combination of EWS shRNA plasmid (0.5 μg/ml) transfection and 100 μM TFL treatment worked superb for the highest reduction in cell viability in SK-N-MC cell line (Figure 2A) as well as in RD-ES cell line (Figure 2B). Therefore, we decided to employ EWS shRNA plasmid (0.5 μg/ml) transfection and 100 μM TFL treatment alone and in combination for controlling growth of Ewing’s sarcoma cells in all subsequent experiments. We also investigated a pharmacological inhibitor of EWS (mithramycin) in combination with TFL and found that mithramycin was not as useful as the genetic inhibitor (EWS shRNA plasmid) in reducing the cell viability in human Ewing’s sarcoma cells.

### 3.3. Knockdown of EWS Expression at mRNA and Protein Levels in Cells

**Figure 3:**
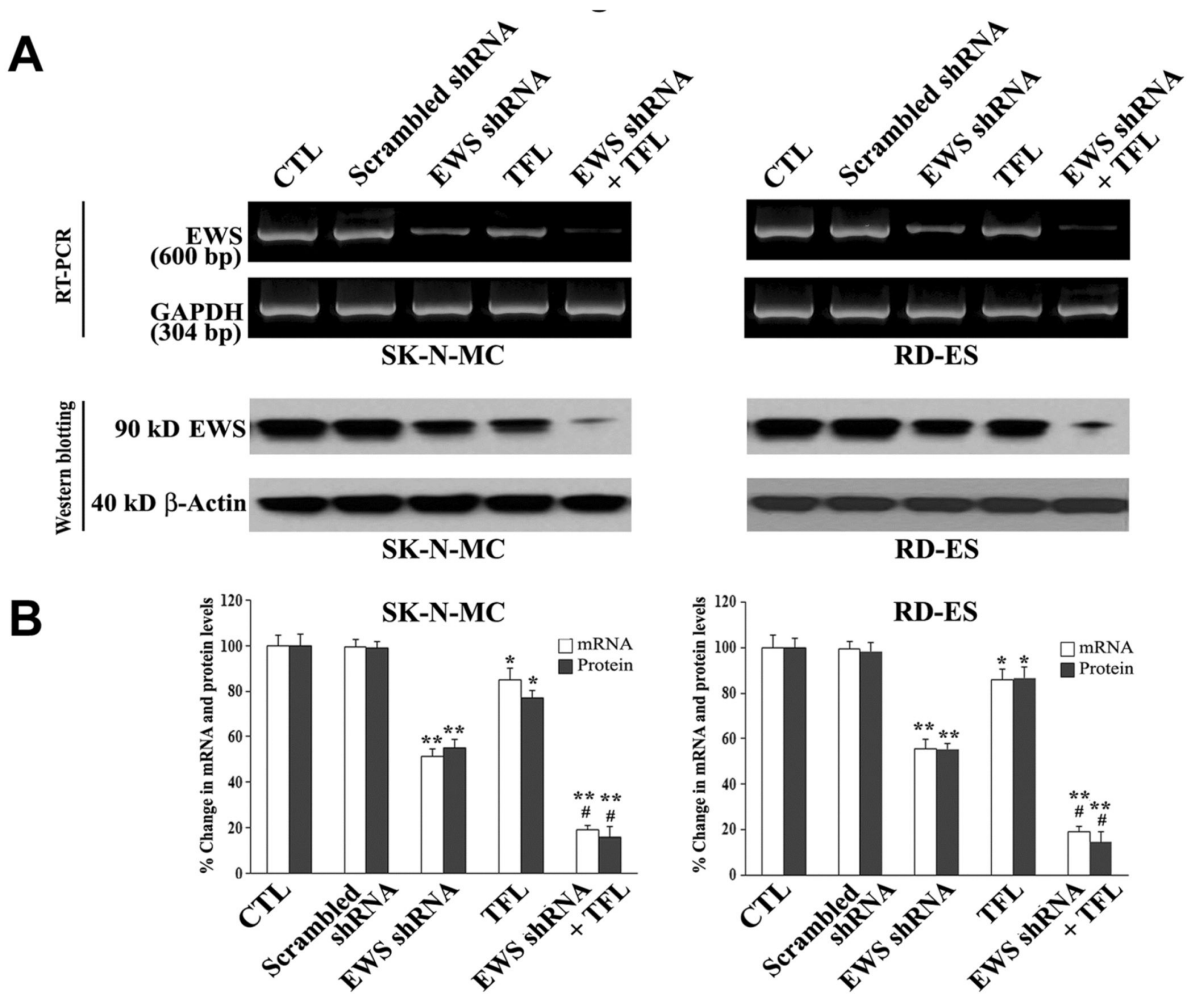
Decreases in expression of EWS at mRNA and protein levels in human Ewing’s sarcoma SK-N-MC and RD-ES cell lines. (A) RT-PCR and Western blotting for changes in expression of EWS at mRNA and protein levels. Expression of GAPDH mRNA or *β*-actin protein was used as internal loading control in RT-PCR or Western blotting, respectively. Treatments: no treatment control (CTL), scrambled shRNA plasmid (0.5 μg/ml) transfection for 72 h, EWS shRNA plasmid (0.5 μg/ml) transfection for 72 h, 100 μM TFL treatment for 24 h, and EWS shRNA palsmid (0.5 μg/ml) transfection for 48 h + 100 μM TFL treatment for last 24 h. (B) Determination of expression of EWS at mRNA and protein levels. Mean values (n = 3) were shown and significant difference between two values was indicated by *P < 0.05 or **P < 0.01 (where monotherapy was compared with CTL) and ^#^*P* < 0.01 (where combination therapy was compared with monotherapy).

We examined the knockdown of EWS expression at mRNA and protein levels in human Ewing’s sarcoma SK-N-MC and RD-ES cell lines after scrambled shRNA plasmid transfection, EWS shRNA plasmid transfection, TFL treatment, and combination of EWS shRNA plasmid transfection and TFL treatment (Figure 3). Our RT-PCR and Western blotting showed the knockdown of EWS expression at mRNA and protein levels, respectively, in both SK-N-MC and RD-ES cell lines after EWS shRNA plasmid transfection, TFL treatment, or both (Figure 3A). RT-PCR and Western blotting were carried out using, respectively, GAPDH mRNA expression and *β*-actin protein expression as the internal controls. Notably, EWS shRNA plasmid transfection was more effective than TFL treatment to knockdown EWS expression at mRNA and protein levels in both cell lines. When compared with untreated control cells, combination of EWS shRNA plasmid transfection and TFL treatment showed the most effective knockdown of EWS expression at mRNA and protein levels in both cell lines. Quantification of mRNA and protein bands by Gel-Pro analyzer software demonstrated greater than 76% knockdown of EWS expression at mRNA and protein levels in both Ewing’s sarcoma cell lines when subjected to combination of EWS shRNA plasmid transfection and TFL treatment (Figure 3B). There was no significant change in EWS expression at mRNA or protein level in the cells due to scrambled shRNA plasmid transfection. Therefore, scrambled shRNA plasmid tranfection was employed as a treated control in all subsequent experiments.

### 3.4. Knockdown of EWS Expression Induced Morphological and Biochemical Features of Differentiation

**Figure 4:**
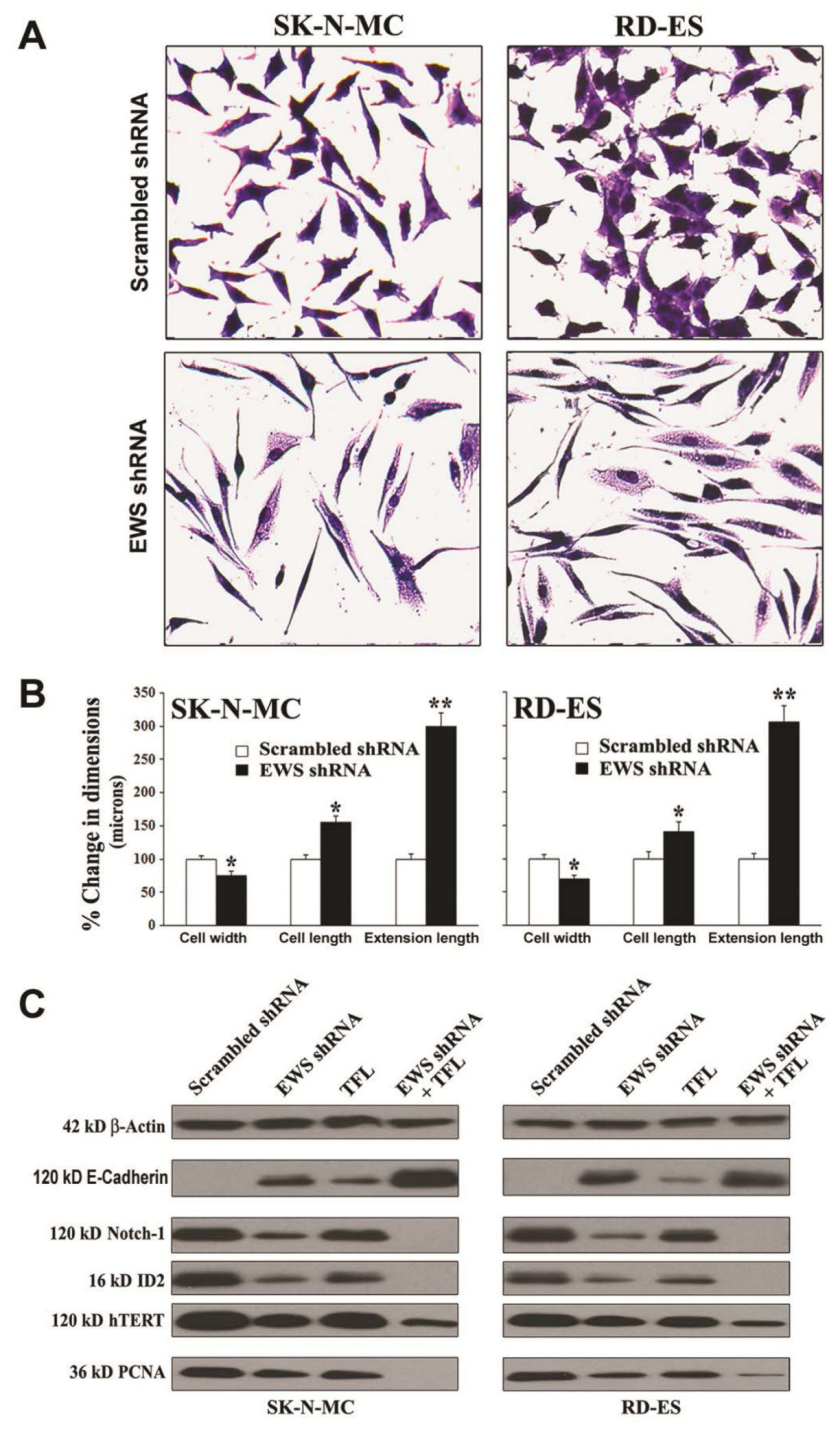
Knockdown of EWS expression inhibited cell proliferation and induced differentiation with modulation of expression of specific molecules in human Ewing’s sarcoma SK-N-MC and RD-ES cell lines. (A) *In situ* methylene blue staining to assess decrease in cell proliferation and increase in morphological features of neuronal differentiation. (B) Measurement of dimensions (cell width, cell length, and extension length) of the differentiated cells. Transfection of cells with scrambled shRNA plasmid (0.5 mg/ml) or EWS shRNA plasmid (0.5 mg/ml) for 72 h. Mean values of three independent experiments were shown. Significant difference between EWS shRNA and scrambled shRNA was indicated by *P < 0.05 or **P < 0.01. (C) Induction of differentiation with modulation of expression of specific molecules. Treatments: scrambled shRNA plasmid (0.5 μg/ml) transfection for 72 h, EWS shRNA plasmid (0.5 μg/ml) transfection for 72 h, 100 μM TFL treatment for 24 h, and EWS shRNA palsmid (0.5 μg/ml) transfection for 48 h + 100 μM TFL treatment for last 24 h. Protein samples were used for Western blotting to examine changes in expression of specific molecules.

It is known that EWS expression is associated with suppression of neuronal differentiation and oncogenic transformation in Ewing’s sarcoma cell lines. So, we sought to examine induction of neuronal differentiation and reduction in oncogenic transformation in Ewing’s sarcoma SK-N-MC and RD-ES cell lines following knockdown of EWS expression (Figure 4). First, we monitored and measured the morphological features of neuronal differentiation in the cells after EWS shRNA plasmid transfection (Figure 4A). The morphological features of neuronal differentiation were revealed in both human Ewing’s sarcoma cell lines using the *in situ* methylene blue staining and light microscopy (Figure 4A). Knockdown of EWS expression by EWS shRNA plasmid transfection very effectively induced morphological features of neuronal differentiation such as small and retracted cell bodies having thin elongated and branched neurite extensions. The scrambled shRNA plasmid transfection did not induce any of these morphological features in the cells. Our measurements of the cell width, cell length, and extension length showed that knockdown of EWS expression by EWS shRNA plasmid transfection significantly diminished the cell width but increased the cell length as well as the extension length, when compared with the cells transfected with the scrambled shRNA plasmid (Figure 4B). Then, we performed Western blotting to examine the alterations in expression of biochemical markers of differentiation and proliferation in the cells (Figure 4C). EWS shRNA plasmid transfection and TFL treatment increased expression of E-cadherin (a tight junction protein), which served as the prominent biochemical marker of differentiation in the cells. Differentiation was also confirmed from the inhibition of expression of Notch-1, ID2 (a member of the inhibitor of differentiation family), and hTERT (catalytic subunit of human telomerase) following knockdown of EWS expression and TFL treatment. Cells put a brake on proliferation before induction of differentiation. Therefore, we confirmed that induction of differentiation was associated with almost complete inhibition of expression of PCNA, a cell proliferation marker, in the cells after knockdown of EWS expression and TFL treatment.

### 3.5. Combination of EWS shRNA Plasmid Transfection and TFL Treatment Prevented Cell Migration and Decreased Molecular Markers of Cell Survival, Angiogenesis, and Invasion

**Figure 5:**
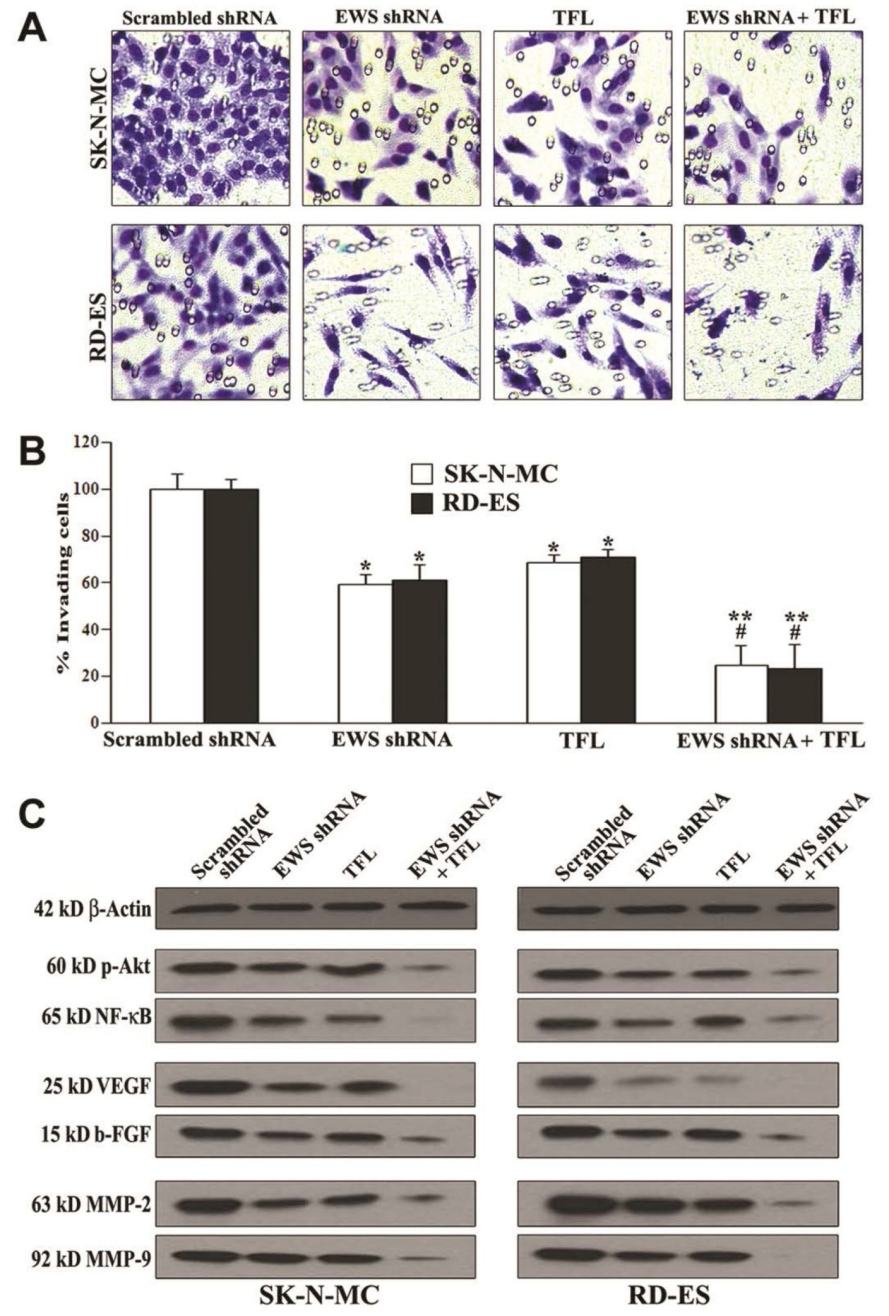
Decreases in cell migration, survival, angiogenesis, and invasion in human Ewing’s sarcoma SK-N-MC and RD-ES cell lines. Treatments: scrambled shRNA plasmid (0.5 μg/ml) transfection for 72 h, EWS shRNA plasmid (0.5 μg/ml) transfection for 72 h, 100 μM TFL treatment for 24 h, and EWS shRNA palsmid (0.5 μg/ml) transfection for 48 h + 100 μM TFL treatment for last 24 h. (A) Cell migration assay. Cells (1 × 10^5^) were seeded for migration through the matrigel coated membrane of tranwell insert following incubation at 37°C in presence of 5% CO_2_ and full-humidity for 48 h. The membranes were collected, stained, and photographed under the light microscope. (B) Quantitation of matrigel invaded cells underneath the membrane. Mean values (n = 3) were shown and significant difference between two values was indicated by *P < 0.05 or **P < 0.01 (where monotherapy or combination therapy was compared with scrambled shRNA) and ^#^*P* < 0.01 (where combination therapy was compared with monotherapy). (C) Western blotting to examine decreases in expression of specific molecules involved in cell survival (p-Akt and NF-*κ*B), angiogenesis (VEGF and b-FGF), and invasion (MMP-2 and MMP-9).

We examined whether knockdown of EWS expression could potentiate TFL in inhibition of migration of human Ewing’s sarcoma SK-N-MC and RD-ES cells through the polycarbonate membranes and we also examined the alterations in expression of molecular markers of survival, angiogenesis, and invasion in the cells Figure 5). Ewing’s sarcoma cells underneath the polycarbonate membrane that could migrate through matrigel were stained and examined using the light microscopy (Figure 5A). The scrambled shRNA plasmid transfection did not prevent Ewing’s sarcoma cells from migration through the matrigel coated polycarbonate membrane. Only knockdown of EWS expression or TFL treatment substantially inhibited cell migration. Most importantly, combination of EWS shRNA plasmid transfection and TFL treatment drastically inhibited cell migration through matrigel coated polycarbonate membrane (Figure 5A). We determined the amounts of cells migration through the transwell polycarbonate membranes and found that combination of knockdown of EWS expression and TFL treatment most significantly inhibited migration of Ewing’s sarcoma cells (Figure 5B). Thereafter, we employed Western blotting to demonstrate that knockdown of EWS expression and TFL treatment most effectively down regulated the molecular markers of cell survival (p-Akt and NF-*κ*B), angiogenesis (VEGF and b-FGF), and invasion (MMP-2 and MMP-9) in Ewing’s sarcoma SK-N-MC and RD-ES cells (Figure 5C).

### 3.6. Combination of EWS shRNA Plasmid Transfection and TFL Treatment Removed DNA Methylation from p53 Promoter to Promote Expression of p53 and Pro-Apoptotic Proteins

**Figure 6:**
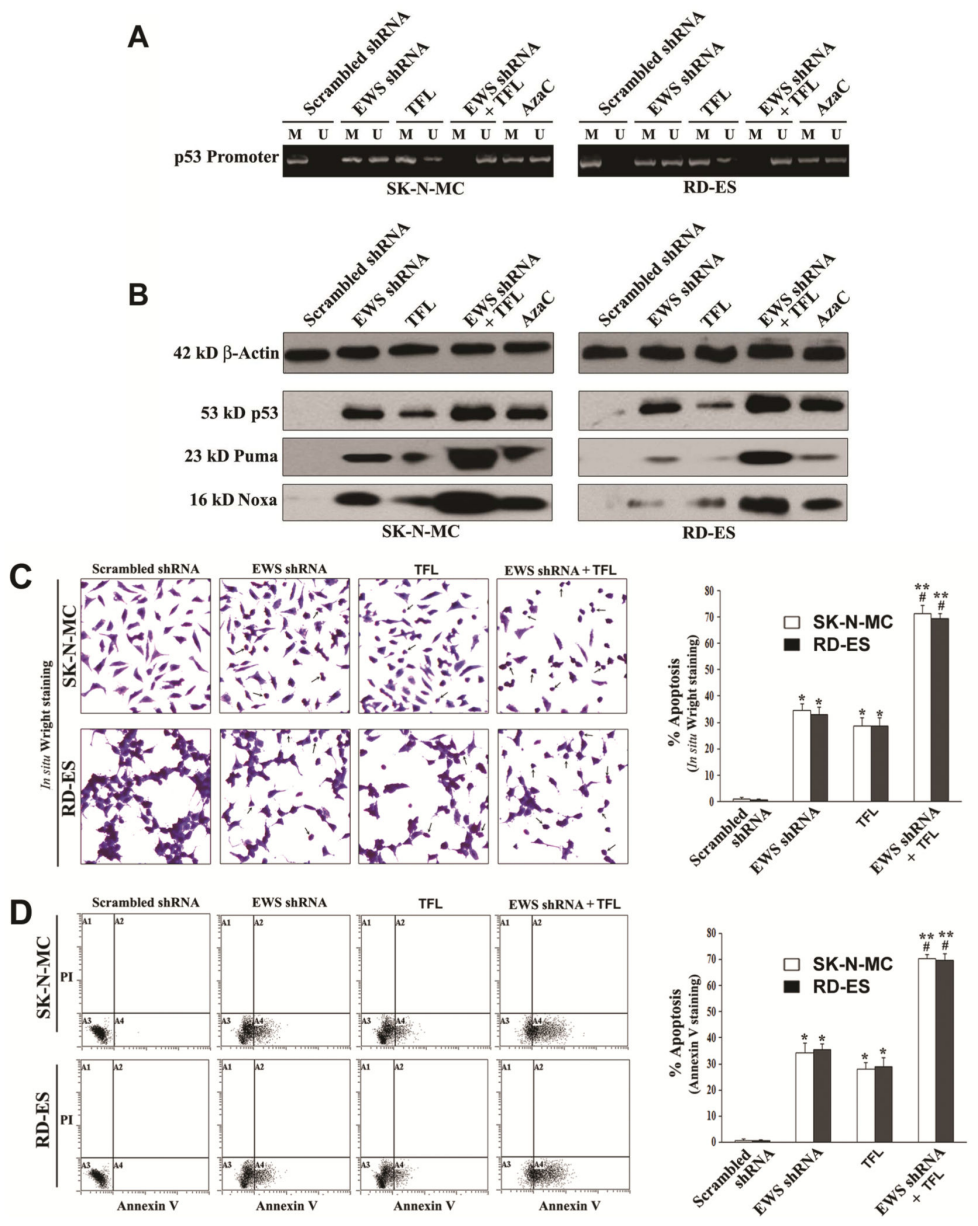
Decrease in DNA methylation at the p53 promoter region promoted apoptosis in human Ewing’s sarcoma SK-N-MC and RD-ES cell lines. Treatments: scrambled shRNA plasmid (0.5 μg/ml) transfection for 72 h, EWS shRNA plasmid (0.5 μg/ml) transfection for 72 h, 100 μM TFL treatment for 24 h, EWS shRNA palsmid (0.5 μg/ml) transfection for 48 h + 100 μM TFL treatment for last 24 h, and 5 μM AzaC for 72 h. (A) MSP analysis of the p53 gene promoter region. The bisulfite-treated genomic DNA samples were used for MSP amplification of the human p53 promoter region using the MSP primers specific for either methylated (M pair) or unmethylated (U pair) DNA. (B) Western blotting to examine expression of the tumor suppressor protein p53 and the pro-apoptotic proteins Puma and Noxa. (C) *In situ* Wright staining for detection and determination of morphological features of apoptosis. (D) Annexin V-FITC/PI staining followed by flow cytometery for detection and determination of a biochemical feature of apoptosis. Mean values (n = 3) were shown and significant difference between two values was indicated by *P < 0.05 or **P < 0.01 (where monotherapy or combination therapy was compared with scrambled shRNA) and ^#^*P* < 0.01 (where combination therapy was compared with monotherapy).

Methylation of DNA at the p53 promoter region is an impediment for expression of this acclaimed tumor suppressor protein in different tumors. Therefore, unmethylation of DNA at the p53 promoter region is an important goal in increasing its expression and tumor suppressor function in promoting expression of other prominent pro-apoptotic proteins such as Puma (p53 upregulated mediator of apoptosis) and Noxa. We examined the effects of EWS shRNA plasmid transfection and TFL treatment on the status of methylation of DNA at the p53 promoter region and protein expression of p53, Puma, and Noxa that could promote apoptosis in both Ewing’s sarcoma SK-N-MC and RD-ES cell lines (Figure 6). We used the bisulfite-treated DNA as template for MSP amplification of the human p53 promoter region using MSP primers specific for either methylated (M pair) or unmethylated (U pair) DNA (Figure 6A). It should be reiterated that amplification with M, U, and both primers indicated methylation, unmethylation, and partial methylation, respectively. The scrambled shRNA plasmid transfection did not remove methylation of DNA from the p53 promoter region. But EWS shRNA plasmid transfection or TFL treatment did partially reduce the methylation of DNA at the p53 promoter region in both cell lines. Importantly, combination of EWS shRNA plasmid transfection and TFL treatment caused complete unmethylation of DNA at the p53 promoter region. We also tested efficacy of AzaC, an inhibitor of DNA methyalation, as positive control for unmethylation of DNA and found that AzaC caused partial unmethylation of DNA at the p53 promoter region in both cell lines. Unmethylation of DNA at the p53 promoter region increased expression of p53 protein, as we demonstrated by Western blotting (Figure 6B). Increase in expression of p53 was correlated with the increases in expression of the pro-apoptotic proteins Puma and Noxa in both Ewing’s sarcoma cell lines.

### 3.7. Combination of EWS shRNA Plasmid Transfection and TFL Treatment Induced Morphological and Biochemical Features of Apoptosis in Ewing’s Sarcoma Cell Lines

The most important goal of an anti-cancer therapy is the induction of apoptotic death in cancer cells. Therefore, we examined whether EWS shRNA plasmid transfection and TFL treatment could induce morphological (Figure 6C) and biochemical (Figure 6D) features of apoptosis in both Ewing’s sarcoma SK-N-MC and RD-ES cell lines. After transfection and treatment, application of the *in situ* Wright staining showed the characteristic morphological features of apoptosis including shrinkage of cell volume, nuclear fragmentation, chromatin condensation and fragmentation, and membrane attached apoptotic bodies (Figure 6C). The scrambled shRNA plasmid transfection (treated control) did not induce any notable morphological features of apoptosis. The arrows were used to indicate the apoptotic cells with at least one of the characteristic morphological changes of apoptosis, which occurred extensively when cells were subjected to combination of EWS shRNA plasmid transfection and TFL treatment. Quantification of apoptotic cells on the basis of *in situ* Wright staining showed that combination of EWS shRNA plasmid transfection and TFL treatment caused the highest amounts of apoptosis in both Ewing’s sarcoma cell lines (Figure 6C). The well-known early biochemical feature of apoptosis is externalization of membrane phospholipids that can be readily detected by Annexin V-FITC staining of the cells. We demonstrated the induction of this early biochemical feature of apoptosis in the cells by flow cytometry (Figure 6D). Annexin V-FITC/PI staining followed by flow cytometry showed that Annexin V-FITC positive apoptotic populations (quadrant A4) were increased moderately in both Ewing’s sarcoma cell lines after EWS shRNA plasmid transfection or TFL treatment alone. But combination of EWS shRNA plasmid transfection and TFL treatment very dramatically increased Annexin V-FITC positive apoptotic populations (quadrant A4) in both cell lines. We determined percentages of apoptotic cells demonstrating clearly that combination of EWS shRNA plasmid transfection and TFL treatment caused the highest increases in induction of apoptotic death in both Ewing’s sarcoma cell lines (Figure 6D).

### 3.8. Combination of EWS shRNA Plasmid Transfection and TFL Treatment Induced Extrinsic and Intrinsic Caspase Cascades for Apoptosis in Ewing’s Sarcoma Cell Lines

**Figure 7:**
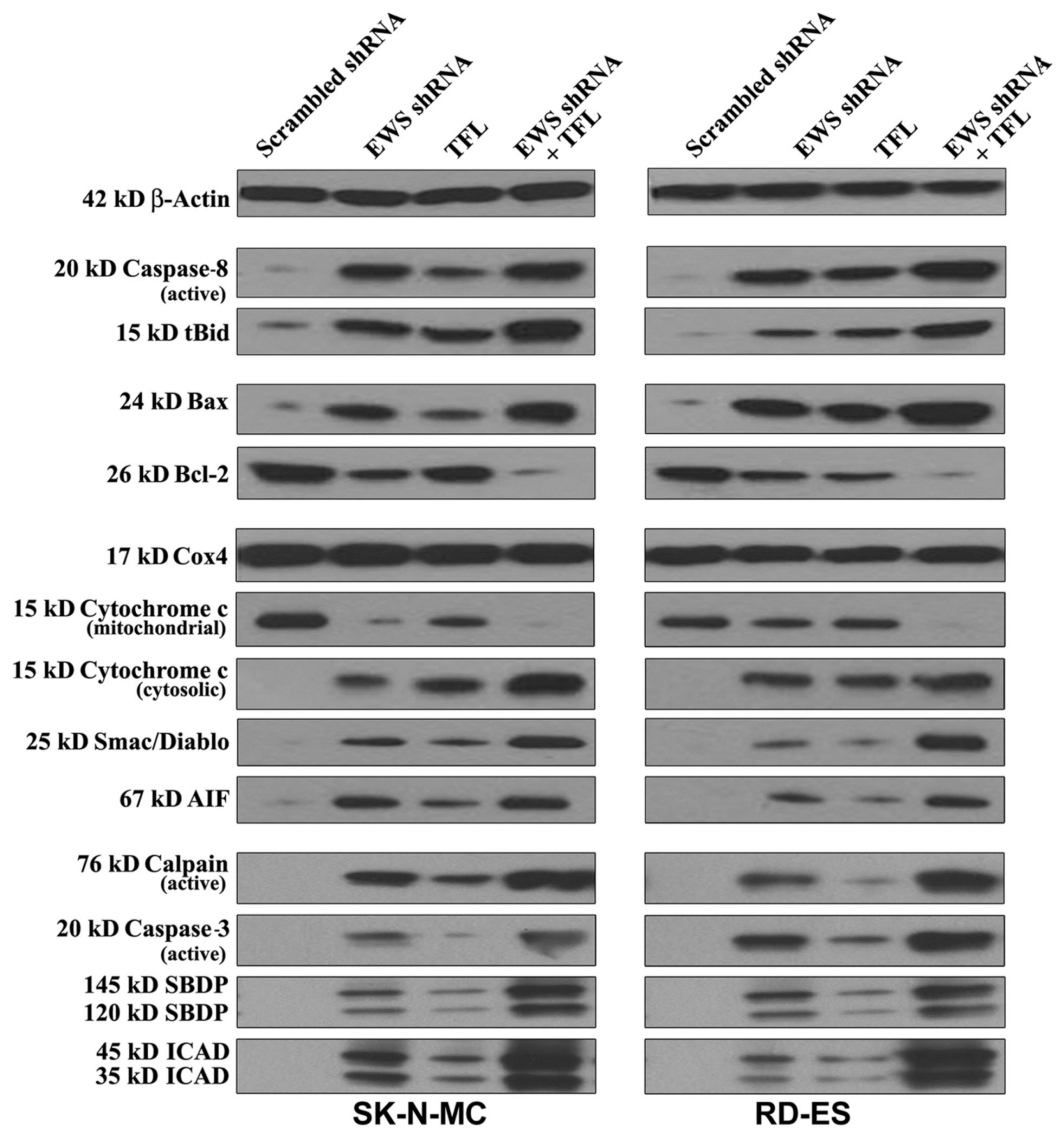
Western blotting to examine modulation of expression of molecules involved in induction of apoptosis in human Ewing’s sarcoma SK-N-MC and RD-ES cell lines. Treatments: scrambled shRNA plasmid (0.5 μg/ml) transfection for 72 h, EWS shRNA plasmid (0.5 μg/ml) transfection for 72 h, 100 μM TFL treatment for 24 h, and EWS shRNA palsmid (0.5 μg/ml) transfection for 48 h + 100 μM TFL treatment for last 24 h.

To reveal the molecular mechanisms for induction of both extrinsic and intrinsic pathways of apoptosis, we performed Western blotting with the protein samples from human Ewing’s sarcoma SK-N-MC and RD-ES cell lines following scrambled shRNA plasmid transfection, EWS shRNA plasmid transfection, TFL treatment, and combination of EWS shRNA plasmid transfection and TFL treatment (Figure 7). Uniform expression of *β*-actin served as a loading control for cytosolic protein samples in Western blotting. EWS shRNA plasmid transfection or TFL treatment moderately induced extrinsic pathway of apoptosis as evidenced from activation of caspase-8 in both cell lines. Combination of EWS shRNA plasmid transfection and TFL treatment most effectively increased activation of caspase-8. Proteolytic activity of caspase-8 cleaved Bid to tBid, which could be translocated to mitochondria for potentiation of intrinsic pathway of apoptosis (Figure 7). The Bcl-2 family proteins play important roles in controlling mitochondrial permeability for induction or inhibition of intrinsic pathway of apoptosis. Combination of EWS shRNA plasmid transfection and TFL treatment caused the highest increase in expression of pro-apoptotic Bax protein and decrease in expression of anti-apoptotic Bcl-2 protein, which could result in increase in the Bax: Bcl-2 ratio for increasing mitochondrial permeability in both Ewing’s sarcoma cell lines. We used uniform expression of Cox4 as a loading control for mitochondrial protein samples in Western blotting (Figure 7). Combination of EWS shRNA plasmid transfection and TFL treatment caused the highest increase in mitochondrial release of cytochrome c, Smac/Diablo, and AIF into the cytosol to trigger mitochondrial caspase-dependent and caspase-independent pathways of apoptosis. We found that combination of EWS shRNA plasmid transfection and TFL treatment most effectively increased the activation and activity of calpain and caspase-3 in both cell lines (Figure 7). Proteolytic activities of calpain and caspase-3 produced calpain-specific 145 kD SBDP and caspase-3-specific 120 kD SBDP, respectively. Further, caspase-3 activity increased ICAD fragmentation, moderately due to monotherapy and mostly due to combination therapy, in both cell lines. Following ICAD fragmentation, release of CAD from the CAD/ICAD complex could translocate CAD to the nucleus for genomic DNA fragmentation.

### 3.9. Combination of EWS shRNA Plasmid Transfection and TFL Treatment Decreased Tumor Growth in Ewing’s Sarcoma Xenografts

**Figure 8:**
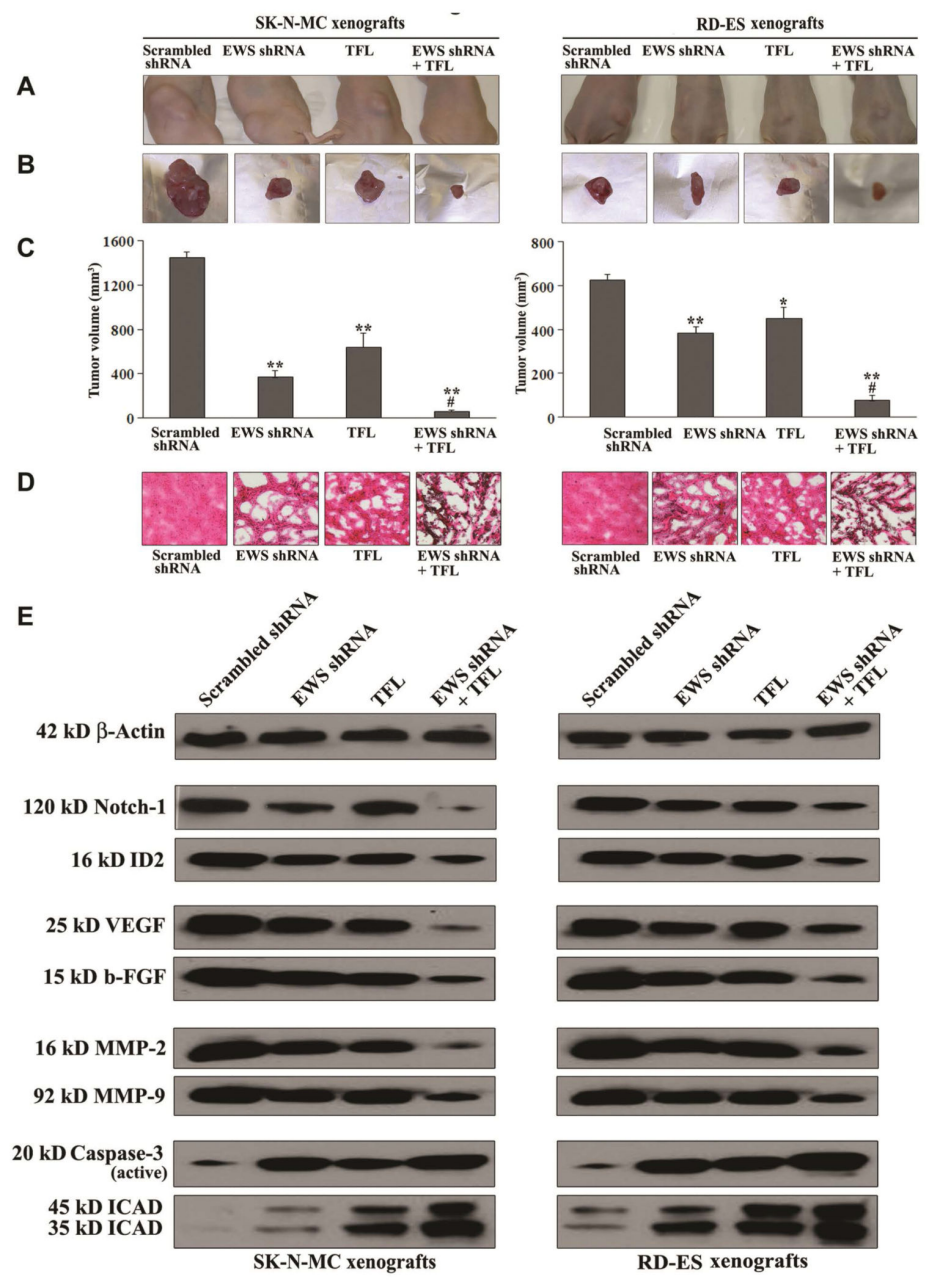
Regression of human Ewing’s sarcoma SK-N-MC and RD-ES xenografts with modulation of expression of various molecules. Mice with xenografts were treated for 2 weeks. Treatments on alternate days: plasmid vector carrying scrambled shRNA cDNA (50 μg DNA/injection/mouse), plasmid vector carrying EWS shRNA cDNA (50 μg DNA/injection/mouse), TFL (20 μg/injection/mouse), and plasmid vector carrying EWS shRNA cDNA (50 μg DNA/injection/mouse) plus TFL (20 μg/injection/mouse). We used 6 animals per group. Significant difference between two values was indicated by *P < 0.05 or **P < 0.01 (where monotherapy or combination therapy was compared with scrambled shRNA) and ^#^*P* < 0.01 (where combination therapy was compared with monotherapy). (A) Mice with SK-N-MC and RD-ES xenografts; (B) Representative tumors; (C) Tumor volume; (D) histopathological changes; and (E) Western blotting to examine modulation of expression of molecules involved in induction of differentiation (Notch-1 and ID2), angiogenesis (VEGF and b-FGF), invasion (MMP-2 and MMP-9), and apoptosis (caspase-3 and ICAD) after the treatments.

We used human Ewing’s sarcoma SK-N-MC and RD-ES cells for development of xenografts in nude mice, which were then subjected to scrambled shRNA plasmid transfection, EWS shRNA plasmid transfection, TFL treatment, and combination of EWS shRNA plasmid transfection and TFL treatment for assessment of alterations in tumor growth, histopathology, and molecular markers (Figure 8). Representative tumors from all treatment groups were shown in the animals (Figure 8A) and also after surgical removal from the animals (Figure 8B). Comparison with the scrambled shRNA plasmid transfection showed that EWS shRNA plasmid transfection or TFL treatment alone reduced tumor growth significantly (Figure 8C). Importantly, combination of EWS shRNA plasmid transfection and TFL treatment showed the highest efficacy in reducing tumor volume and also more significant efficacy than EWS shRNA plasmid transfection or TFL treatment alone in decreasing tumor volume (Figure 8C). Following all the treatments, we performed H & E staining of the tumor sections for examination of alterations in cell proliferation and cell death (Figure 8D). We found that scrambled shRNA plasmid transfection group maintained characteristic tumor growth, EWS shRNA plasmid transfection or TFL treatment alone inhibited cell proliferation and induced cell death to some extent, but combination of EWS shRNA plasmid transfection and TFL treatment caused the most dramatic inhibition of cell proliferation and induction of massive cell death in both Ewing’s sarcoma xenograft models (Figure 8D). So, combination of EWS shRNA plasmid transfection and TFL treatment was a better strategy than either agent alone in controlling growth of human Ewing’s sarcomas *in vivo*.

### 3.10. Combination of EWS shRNA Plasmid Transfection and TFL Treatment Induced Differentiation, Inhibited Angiogenesis and Invasion, and Induced Apoptosis in Ewing’s Sarcoma Xenografts

We performed Western blotting to examine the effects of scrambled shRNA plasmid transfection, EWS shRNA plasmid transfection, TFL treatment, and combination of EWS shRNA plasmid transfection and TFL treatment on the molecular events for induction of differentiation, inhibition of angiogenesis and invasion, and induction of apoptosis in human Ewing’s sarcoma SK-N-MC and RD-ES xenografts (Figure 8E). We used almost uniform expression of *β*-actin as a protein loading control in Western blotting. Induction of differentiation in tumor cells could be triggered by down regulation of Notch-1 and ID2, both of which were indeed down regulated very clearly due to combination of EWS shRNA plasmid transfection and TFL treatment in Ewing’s sarcoma SK-N-MC and RD-ES xenografts. Also, the levels of expression of the well-known pro-angiogenic molecules (VEGF and b-FGF) and pro-invasive molecules (MMP-2 and MMP-9) were highly inhibited following combination of EWS shRNA plasmid transfection and TFL treatment in Ewing’s sarcoma xenografts. Activation and activity of caspase-3, the final executioner of apoptosis, could confirm the efficacy of a therapeutic strategy for induction of apoptotic cell death in the xenografts. Our Western blotting revealed that combination of EWS shRNA plasmid transfection and TFL treatment most effectively caused activation of caspase-3 that showed the highest proteolytic activity in ICAD fragmentation in both Ewing’s sarcoma SK-N-MC and RD-ES xenografts.

## 4. Discussion

Our current investigation showed that sequential knockdown of EWS expression and TFL treatment induced differentiation and inhibited cell migration, angiogenesis, and invasion leading to induction of apoptosis in human Ewing’s sarcoma SK-N-MC and RD-ES cells having EWS overexpression. Because EWS expression plays important roles in the pathogenesis of Ewing’s sarcoma [6], it is a prime therapeutic target for controlling this malignancy. As an aberrant transcription factor, it regulates expression of a variety of genes [31] [32] that modulate important functions such as differentiation, cell proliferation, angiogenesis, and apoptosis in tumor cells. We confirmed constitutive expression of EWS protein in Ewing’s sarcoma SK-N-MC and RD-ES cells and demonstrated modulation of molecular components for induction of differentiation and inhibition of angiogenesis and invasion leading to apoptosis in these malignant cells in culture and animal models after knockdown of EWS expression and TFL treatment.

In the present study, we successfully employed EWS shRNA plasmid to knockdown EWS mRNA and thereby its protein levels in two human Ewing’s sarcoma SK-N-MC and RD-ES cell lines in cultures. EWS shRNA plasmid transfection resulted in more than 40% knockdown and this transfection followed by TFL treatment resulted in more than 80% knockdown of EWS at the mRNA and protein levels, indicating the superb efficacy of this combination therapeutic strategy for suppression of EWS expression in SK-N-MC and RD-ES cell lines.

We found that EWS shRNA plasmid transfection down regulated EWS and induced differentiation resulting in significant decrease in cell width and significant increases in the cell length and extension length, all of which indicated a morphological transition to neuronal phenotype. We showed that transition to this phenotype was accompanied by increase in expression of E-cadherin, which was previously shown to promote cell differentiation [33], and also substantial decreases in expression of not only EWS but also Notch-1 and Id2, which were previously shown to promote dedifferentiation of the cells [34]. Induction of differentiation is known to be correlated with repression of cell proliferation markers such as hTERT and PCNA [35]. Combination of EWS shRNA plasmid transfection and TFL treatment drastically down regulated hTERT and PCNA indicating that inhibition of cell proliferation promoted differentiation of Ewing’s sarcoma cells.

Distinct ability of the tumor cells to infiltrate through the extracellular matrix makes it very difficult to treat Ewing’s sarcoma using surgery and radiation. The dislodgment of tumor cells from the primary site and their subsequent migration to normal adjacent tissues is a characteristic feature of Ewing’s sarcoma. In the present study, we performed cell migration assay to measure the ability SK-N-MC and RD-ES cell lines that could migrate and then pass through the matrigel before and after EWS shRNA plasmid transfection or/and TFL treatment. Notably, combination therapy resulted in upto 80% inhibition in migration ability of Ewing’s sarcoma cells. We did not use any chemoattractant in the medium placed underneath the membrane. This indicated that the treated tumor cells lost the ability to secrete the required amount of proteolytic enzymes such as MMPs to degrade the matrix and pass through the membrane.

Phosphorylation of Akt (p-Akt) triggers Akt kinase activity, which has high potential to deregulate cell cycle, induce cell proliferation, avoid apoptosis, and stimulate cell survival through upregulation of NF-*κ*B [36]. Another subsequent study also reports that p-Akt promotes cell survival and proliferation via activation of NF-*κ*B signaling [37]. Activation of NF-*κ*B protein complex controls transcription of DNA in nucleus. Deregulation of NF-*κ*B is linked to pathogenesis of cancer and many inflammatory and autoimmune diseases. Combination of EWS shRNA plasmid transfection and TFL treatment most effectively decreased expression of p-Akt, and NF-*κ*B in SK-N-MC and RD-ES cell lines.

Angiogenesis, which is the formation of new blood vessels from pre-existing ones, plays an important role in tumor progression and metastasis. The mechanism of angiogenesis is primarily mediated by hypoxia through chronic activation of the hypoxia-inducible factor pathway leading to production of VEGF and b-FGF [38] [39]. We observed that human Ewing’s sarcoma cells subjected to EWS shRNA plasmid transfection and TFL treatment failed to produce the potent angiogenic factors VEGF and b-FGF, indicating efficacy of this combination therapy in blocking angiogenesis. Our combination therapy also markedly reduced expression of MMPs such as MMP-2 and MMP-9, which would otherwise play prominent role in cell migration through proteolytic degradation of extracellular matrix.

The restoration or promotion of the tumor suppressor function of p53 protein is an important goal in the development of new therapeutic strategies. The tumor suppressor function of p53 is triggered by a variety of oncogenic proteins including Myc, Ras, E1A, and *β*-catenin [40]. Introduction of CpG methylation at the human p53 promoter region down regulated the transcriptional activity of p53 [41], indicating the necessity of unmethylation at the human p53 promoter region for promotion of its transcription and tumor suppressor function. We observed that combination of EWS shRNA plasmid transfection and TFL treatment caused unmethylation at the p53 promoter to promote expression of p53 protein. Puma (p53 upregulated modulator of apoptosis) is a pro-apoptotic, BH3-only, Bcl-2 family member that regulates p53 dependent and independent apoptosis [42]. A recent study has shown that Puma is upregulated following treatment with tamoxifen to induce apoptosis in breast cancer cells [43]. Puma promotes apoptosis by inhibiting the interaction of anti-apoptotic proteins such as Bcl-2 and Bcl-xL with their pro-apoptotic counterparts such as Bax and Bak [44]. We revealed an upregulation of Puma after our combination therapy, suggesting potentiation of pro-apoptotic machinery in human Ewing’s sarcoma cells. Noxa is another BH3-only pro-apoptotic protein, which has been shown to induce apoptosis by binding with Bcl-xL and sequestering it; thus Noxa inhibits the pro-survival attributes of Bcl-xL [45]. We showed upregulation of Noxa in human Ewing’s sarcoma cells after EWS shRNA plasmid transfection and TFL treatment.

Our morpholical and biochemical staining assays demonstrated that EWS shRNA plasmid transfection and TFL treatment significantly increased the apoptotic features in SK-N-MC and RD-ES cell lines. It has previously been reported that plant-derived flavonoids induce death receptor-mediated caspase-8 activation followed by caspase-3 activation for induction of apoptosis in tumor cells [14]. Therefore, we analyzed the activation and activity of caspase-8 to confirm activation of the receptor-mediated pathway of apoptosis in human Ewing’s sarcoma cells. Active caspase-8 shows its proteolytic activity in the cleavage of Bid to tBid, which then translocates to mitochondrial membrane to aid mitochondrial release of several pro-apoptotic factors including cytochrome c, Smac/Diablo, and AIF into the cytosol to promote apoptosis [33] [46]. Thus, translocation of tBid to mitochondria provides a link between death receptor-mediated (extrinsic) and mitochondria-mediated (intrinsic) pathways of apoptosis [47]. Also, increase in pro-apoptotic Bax and decrease in anti-apoptotic Bcl-2 could result in increase in Bax:Bcl-2 ratio to trigger the mitochondrial release of the pro-apoptotic molecules into the cytosol [48]. Cytosolic cytochrome c binds to apoptosis-activating-factor-1 (Apaf-1) to produce apoptosome leading to sequential activation of caspase-9 and caspase-3 for caspase-dependent apoptosis [33] [49]. Caspases play significant roles in induction of apoptosis in cancer cells following treatment with anti-cancer agents. Proteolytic activities of calpain and caspase-3 are known to simultaneously act on the same *α*-spectrin as substrate to produce specific SBDPs and thereby facilitate the apoptotic death in cancer cells [50]. Caspae-3 activity alone can cleave ICAD to promote translocation of CAD to the nucleus for genomic DNA fragmentation, the final step in apoptosis. We found that EWS shRNA plasmid transfection and TFL treatment most effectively modulated the expression and activity of molecular components of both the extrinsic and intrinsic pathways to take apoptotic process to the final step in human Ewing’s sarcoma SK-N-MC and RD-ES cells.

Most importantly, our combination therapy worked very efficiently in the animal models of Ewing’s sarcoma to reduce tumor volume drastically due to histopathological alterations such as inhibition of cell proliferation and induction of cell death. We also revealed that tumor regression was due to molecular alterations to promote differentiation, inhibit angiogenesis and invasion, and induce apoptosis in Ewing’s sarcoma xenografts in animal models.

## 5. Conclusion

In conclusions, the present study demonstrated that EWS shRNA plasmid transfection and TFL treatment most effectively increased the anti-tumor mechanisms such as induction of differentiation and inhibition of angiogenesis as well as of invasion leading to apoptotic death in human Ewing’s sarcoma SK-N-MC and RD-ES cells in culture and animal models. Thus, results of this investigation demonstrate that the combination of EWS shRNA plasmid transfection and TFL treatment can offer a novel therapeutic strategy for controlling the growth of human Ewing’s sarcoma. This new combined therapeutic strategy has the potential for translation to the clinics for inhibition of growth of Ewing’s sarcoma in humans in the near future.
